# A Quinoxaline 1,4-Dioxide Activates DNA Repair Systems in *Mycobacterium smegmatis*: A Transcriptomic Study

**DOI:** 10.3390/ijms26083689

**Published:** 2025-04-14

**Authors:** Olga B. Bekker, Olesya O. Galanova, Aleksey A. Vatlin, Svetlana G. Frolova, Egor A. Shitikov, Dmitry A. Bespiatykh, Ksenia M. Klimina, Vladimir A. Veselovsky, Rustem A. Ilyasov, Svetlana V. Smirnova, Diana A. Reznikova, Nikita I. Kochetkov, Dmitry A. Maslov, Valery N. Danilenko

**Affiliations:** 1 Laboratory of Bacterial Genetics, Vavilov Institute of General Genetics, Russian Academy of Sciences, 119333 Moscow, Russia; obbekker@mail.ru (O.B.B.); sveta.frolova.1997@bk.ru (S.G.F.); apismell@hotmail.com (R.A.I.); vsmirnov64@mail.ru (S.V.S.); reznikova.da@phystech.su (D.A.R.); samatrixs@gmail.com (N.I.K.); valerid@vigg.ru (V.N.D.); 2 Moscow Center for Advanced Studies, 123592 Moscow, Russia; 3 Institute of Ecology, Peoples’ Friendship University of Russia (RUDN University), 117198 Moscow, Russia; 4 Division of Biomedicine and Genomics, Lopukhin Federal Research and Clinical Center of Physical-Chemical Medicine of Federal Medical Biological Agency, 119435 Moscow, Russia; egorshtkv@gmail.com (E.A.S.); d.bespiatykh@gmail.com (D.A.B.); ppp843@yandex.ru (K.M.K.).; djdf26@gmail.com (V.A.V.); 5 Developmental Neurobiology Laboratory, Koltsov Institute of Developmental Biology of Russian Academy of Sciences, 119334 Moscow, Russia; 6 Faculty of Biotechnology and Fisheries, Moscow State University of Technologies and Management (FCU), 109004 Moscow, Russia; 7 Division of Gastroenterology and Hepatology, Department of Medicine, Stanford University School of Medicine, Stanford, CA 94305, USA; dmaslov@stanford.edu

**Keywords:** *M. smegmatis*, QdNOs, antituberculosis agent, transcriptome

## Abstract

In 2022, the World Health Organization reported that tuberculosis (TB) was the second leading cause of death globally from a single infectious agent following COVID-19. The development of new antitubercular agents with novel mechanisms of action for use in complex TB therapy is considered a key approach to combating TB. In this study, we examined the gene expression profile of *M. smegmatis* when exposed to a promising antituberculosis agent, quinoxaline 1,4-dioxide (QdNO) 7-chloro-2-(ethoxycarbonyl)-3-methyl-6-(piperazin-1-yl)quinoxaline-1,4-dioxide-1 (LCTA-3368). We investigated how the bacterial response changed with different minimum inhibitory concentrations (MIC) (1/4 × MIC, 1/2 × MIC, and 1 × MIC) and durations (30 min and 90 min) of treatment with the drug. Our analysis revealed significant upregulation in genes involved in DNA repair and replication processes, as well as changes in the expression of 95 genes encoding proteins with oxidoreductase activity. We additionally showed that the concentration of reactive oxygen species increases in a dose-dependent manner upon exposure of *M. smegmatis* to LCTA-3368. These findings support the proposed mechanism of antibacterial action of QdNOs, which is associated with the formation of free radicals leading to DNA damage.

## 1. Introduction

According to the World Health Organization (WHO), *Mycobacterium tuberculosis* and tuberculosis (TB) were the world’s second leading cause of death from a single infectious agent in 2022, after coronavirus disease (COVID-19), and caused nearly twice as many deaths as HIV/AIDS. The global number of people newly diagnosed with TB reached 7.5 million in 2022, the highest number reported since WHO began global TB monitoring in 1995. Globally, an estimated 410,000 people (95% UI: 370,000–450,000) developed multidrug-resistant TB (MDR-TB, defined as TB resistant to rifampicin and isoniazid) or rifampicin-resistant TB (RR-TB) in 2022 [1].

Urgent action is required to end the global TB epidemic by 2030, a goal adopted by all United Nations Member States and the WHO. Given the rising incidence of multidrug-resistant TB, the discovery, development, and rapid adoption of new tools, interventions, and treatment strategies are among the key priorities in TB treatment. Additional priorities include the development of a vaccine to reduce the risk of infection and new drug treatments to combat MDR-TB [1].

Quinoxaline 1,4-dioxides (QdNOs) are promising compounds with a broad spectrum of biological properties, including antitumor, antibacterial, antiparasitic, anti-inflammatory, antioxidant, and herbicidal activities [2]. Recent studies have shown that some QdNO derivatives exhibit excellent inhibitory activity against *M. tuberculosis*, highlighting the potential of this scaffold for the development of new anti-TB drugs [3,4,5]. The presence of two N-oxide fragments in the quinoxaline ring contributes to their high biological activity by enabling oxidation processes [6]. Additionally, QdNOs can undergo bioreductive activation under the hypoxic conditions present in TB granulomas, where non-replicating persistent forms of *M. tuberculosis* can survive. This survival mechanism contributes to prolonged treatments and the risk of drug resistance development [7].

In previous work, we identified a lead compound, 7-chloro-2-(ethoxycarbonyl)-3-methyl-6-(piperazin-1-yl)quinoxaline 1,4-dioxide-1 (LCTA-3368), through a screen of a QdNO library using *Mycobacterium smegmatis* [8], a model organism widely employed for screening anti-TB drug candidates [9,10]. LCTA-3368 (Figure S1) demonstrated strong inhibitory activity against both *M. smegmatis* (4 μg/mL) and *M. tuberculosis* (1.25 μg/mL), although its cytotoxicity remains to be evaluated. We found that LCTA-3368 induces unique non-synonymous mutations in a variety of genes in spontaneous drug-resistant *M. smegmatis* mutants, although a clearly defined cellular target has not yet been identified [8]. One proposed mechanism of action for quinoxaline 1,4-dioxide derivatives involves the direct induction of single- and double-stranded DNA breaks in bacteria, which could explain the numerous mutations observed in *M. smegmatis* strains [11].

Transcriptomic studies, including comprehensive RNA sequencing (RNA-seq) analysis, are increasingly becoming a powerful tool for elucidating additional mechanisms of action of antimicrobials, including antituberculosis drugs, by examining the bacterial transcriptional response to drug exposure [12]. In our previous study, we employed transcriptomic analysis to investigate the impact of imidazo tetrazines on iron metabolism, a set of compounds for which a biotarget could not be identified using the classical approach of generating spontaneous drug-resistant mutants [13].

In this study, we describe the transcriptomic profile of *M. smegmatis* treated with LCTA-3368 in a time-dependent (30 min and 90 min) and dose-dependent (1/4 × MIC, 1/2 × MIC, and 1 × MIC) manner to gain insight into the gradual changes in the bacterial response to this drug. We observed significant alterations in the expression of genes involved in DNA repair and replication processes.

## 2. Results

### 2.1. Whole-Transcriptome Analysis

A total of 32 RNA-seq libraries were generated to comprehensively characterize the transcriptional profile of *M. smegmatis* following LCTA-3368 treatment at different concentrations and timepoints, yielding 167,144,850 raw reads. After the removal of low-quality and adapter sequences, 165,637,398 clean reads were retained, representing an average retention rate of 98.75–99.96% of the raw reads. The average length of bases mapped per library was 256,321,391 bp.

**Figure 1:**
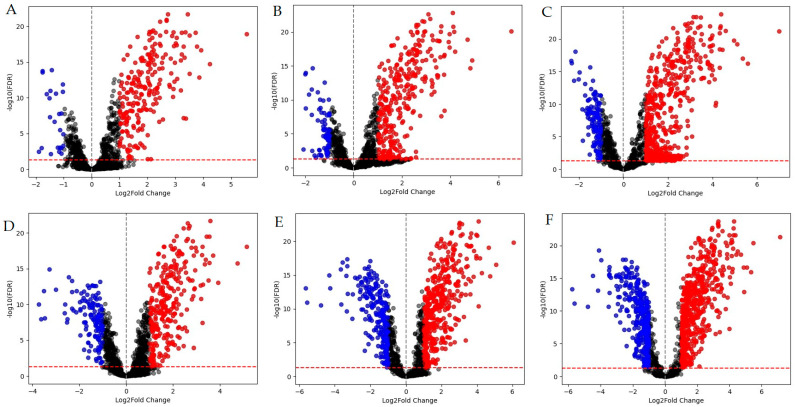
Differentially expressed genes (DEGs) in *M. smegmatis*. Red dots represent positively regulated DEGs (log2FC ≥ 1), while blue dots indicate negatively regulated DEGs (log2FC ≤ −1). Graphs (**A**–**C**) display volcano plots for samples incubated for 30 min, whereas graphs (**D**–**F**) correspond to samples incubated for 90 min in the presence of LCTA-3368 at concentrations of 1/4 × MIC (**A**,**D**), 1/2 × MIC (**B**,**E**), and 1 × MIC (**C**,**F**).

A total of 1223 differentially expressed genes (DEGs) with at least a two-fold change in expression level were identified (Table S1). Among these, 767 genes were upregulated, with 209 of them consistently expressed across all experimental conditions. Conversely, 456 DEGs were downregulated, with five of these consistently expressed at all timepoints and concentrations (Figure 1).

**Figure 2:**
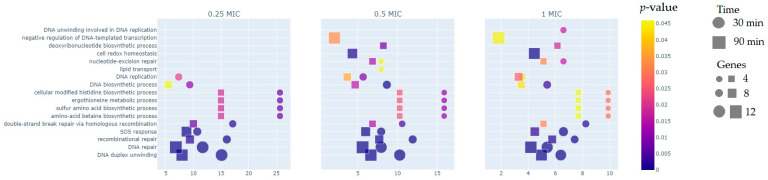
GO analysis of DEGs corresponding to different timepoints and concentrations of LCTA-3368. The Y-axis represents KEGG pathways, while the X-axis represents fold enrichment (calculated as the ratio of input pathway DEGs to the background gene set). The color of the dots indicates the *p*-values of enrichment (*p* < 0.005), the size of the dots corresponds to the number of genes in the pathway, and the shape of the dots represents the treatment time.

The comparison of different MIC groups reveals that more than half of the differentially expressed genes (DEGs) are represented after 90 min of exposure to the test compound, particularly in the 1/2 × MIC and 1 × MIC groups. Regarding the GO analysis (Figure 2), approximately 30% of the DEGs, on average, are associated with the DNA repair pathway (GO:0006281) and the DNA duplex unwinding pathway (GO:0032508) for both 30-min and 90-min exposures across all three groups (1/4 × MIC, 1/2 × MIC, 1 × MIC). At the 90-min timepoint, negative regulation of DNA-templated transcription (GO:0045892) was observed in the 1/2 × MIC and 1 × MIC groups but not in the 1/4 × MIC group. A similar pattern was observed for the nucleotide-excision repair (GO:0006289) and cell redox homeostasis (GO:0045454) pathways. Differential expression of genes in the lipid transport pathway (GO:0006869) was detected only in the 30-min exposure at 1/2 × MIC, while genes related to the DNA unwinding involved in the DNA replication pathway (GO:0006268) were identified exclusively in the 30-min exposure at 1 × MIC.

Since we identified a large number of differentially expressed genes (DEGs), we introduced a dose-dependence criterion to further analyze the results. The primary criterion for dose-dependence is a minimum difference of 0.5 for upregulated genes and −0.5 for downregulated genes in the logarithm of the fold change in base 2 (log2FC) values at MIC, and 1/4 × MIC, 1/2 × MIC, 1 × MIC, with this difference required at each step. We selected DEGs meeting this criterion at both the 30-min and 90-min timepoints, and these will be referred to as “dose-dependent” hereafter. This difference was evaluated separately for the 30-min and 90-min timepoints, as well as simultaneously for both timepoints. Thus, DEGs could be dose-dependent at 30 min, 90 min, or both. An additional criterion is that the log2FC must change in a consistent direction (either increasing or decreasing) with the increasing concentration of LCTA-3368. DEGs satisfying all these conditions are classified as “dose-dependent”.

### 2.2. Functional Groups of Differentially Expressed Genes

The analysis identified 42 DEGs involved in DNA repair, 86 DEGs encoding transcriptional regulators, highlighting the robust response to LCTA-3368, 91 DEGs encoding proteins with oxidoreductase functions, 55 DEGs whose products participate in transport processes, 57 DEGs associated with fatty acid metabolism, amino acid metabolism, and the TCA cycle, and 13 DEGs related to transposases (Table S2).

#### 2.2.1. Reparation Genes

The analysis identified 42 DEGs associated with the DNA repair pathway, encompassing all known DNA repair systems in mycobacteria: double-strand break repair (DSB), homologous recombination (HR), nonhomologous end joining (NHEJ), single-strand annealing (SSA), nucleotide excision repair (NER), and the SOS response system (SOS) (Table S3). All DNA repair-related DEGs were upregulated throughout the experiment, confirming the extensive DNA damage inflicted on bacterial cells by LCTA-3368 [14].

Among the 42 DEGs in Mycobacterium smegmatis, the proteins encoded by 33 DEGs exhibit homology to proteins in Mycobacterium tuberculosis, with sequence similarity ranging from 75% to 98% and differences in amino acid sequence lengths of less than 15% (Table S3). A total of 38 M. smegmatis DEGs have counterparts in the M. tuberculosis genome. However, the amino acid sequences of the primosomal protein PriA (*Rv1402* in *M. tuberculosis* and *MSMEG_1238* in *M. smegmatis*) are not homologous despite sharing the same function. PriA is involved in replication restart in bacteria by unwinding the lagging strand of stalled DNA replication forks. Similarly, RecF, which participates in homologous recombination, shows low homology between *MSMEG_2293* and *Rv0003*. Notably, *M. smegmatis* possesses a gene encoding a *swt1*-family HEPN helicase and an additional gene for a UvrD-like protein, both of which are absent in the *M. tuberculosis* genome. Importantly, the key genes responsible for the functioning of all DNA repair systems in *M. smegmatis* have homologs in *M. tuberculosis*.

**Figure 3:**
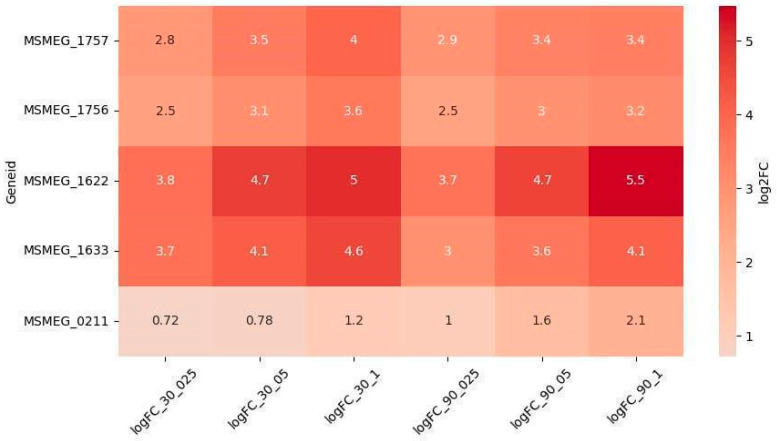
Differentially expressed genes (DEGs) associated with DNA repair systems exhibiting dose-dependent regulation.

Among the DEGs, five genes were upregulated in a dose-dependent manner (Figure 3): *MSMEG_1622* (DNA repair) and *MSMEG_1633* (SOS) were the most significantly upregulated and dose-dependent at both timepoints. *MSMEG_1756* and *MSMEG_1757* (NER) exhibited dose-dependent upregulation at 30 min, while *MSMEG_0211* (NER) showed dose-dependent upregulation at 90 min. Interestingly, the upregulation of *MSMEG_0211* increased at 90 min compared to 30 min, whereas the expression of other dose-dependent DEGs either remained stable or slightly decreased at 90 min.

#### 2.2.2. Oxidoreductases

The largest group of DEGs (N = 91, comprising 69 upregulated and 22 downregulated DEGs) consisted of genes encoding proteins with predicted oxidoreductase activity. These proteins belong to the following families: SDR family, Nitroreductase superfamily, VOC family, LLM class flavin-dependent oxidoreductases, and FAD-binding oxidoreductases. Previous studies have shown that bacterial oxidoreductases may play a role in the metabolic activation of quinoxalines, generating free radicals that can inhibit DNA synthesis and cause DNA damage [15].

**Figure 4:**
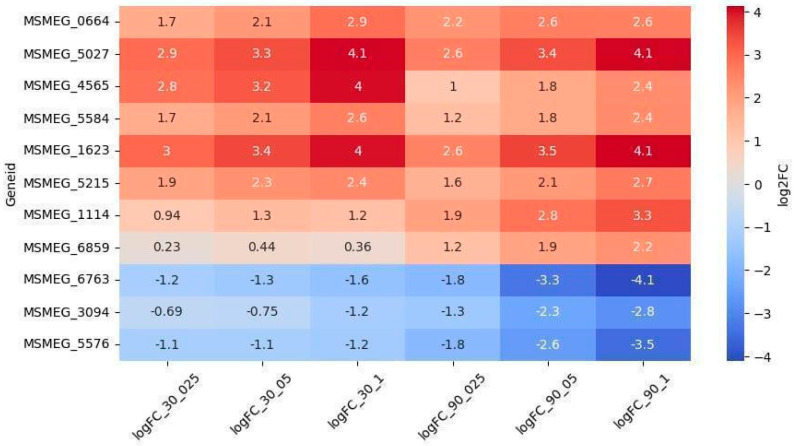
Dose-dependent expression patterns of oxidoreductase-related differentially expressed genes (DEGs).

Among the 69 upregulated genes, 8 exhibited dose-dependent regulation (Figure 4). The genes *MSMEG_5027* (encoding a VOC family protein) and *MSMEG_1623* (encoding an SDR family oxidoreductase) were the most significantly upregulated and the only ones showing dose-dependence at both timepoints. Conversely, the genes *MSMEG_3094*, *MSMEG_5576*, and *MSMEG_6763* were downregulated throughout the experiment and displayed dose dependence at the 90-min timepoint.

#### 2.2.3. Other Genes

**Figure 5:**
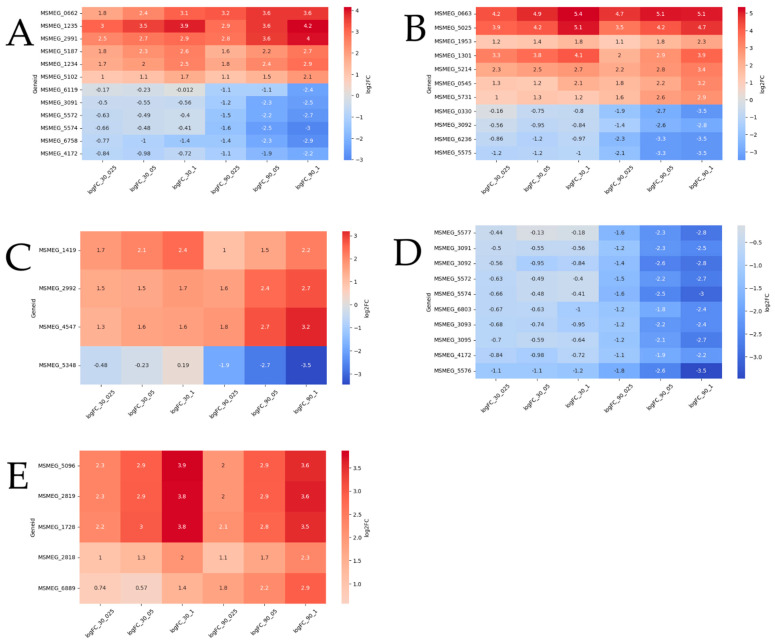
Heatmap illustrating dose-dependent differentially expressed genes (DEGs). Red indicates upregulated DEGs, while blue represents downregulated DEGs. The graphs depict DEGs associated with (**A**) transport activity, (**B**) transcriptional regulator activity, (**C**) fatty acid metabolic activity, (**D**) carbohydrate metabolism and transport, and (**E**) transposase activity.

Differential expression analysis identified 55 transport-related genes, with 23 upregulated and 32 downregulated. Among these, 12 exhibited dose-dependent expression patterns (Figure 5A). Specifically, genes *MSMEG_2991* and *MSMEG_5187* (encoding multidrug efflux MFS transporters), *MSMEG_5102*, *MSMEG_1234*, *MSMEG_0662* (encoding ABC transporters), and *MSMEG_1235* (encoding a SulP transporter) were upregulated in a dose-dependent manner at both timepoints. Additionally, *MSMEG_3091*, *MSMEG_4172*, *MSMEG_5572*, *MSMEG_5574* (encoding carbohydrate ABC transporters), *MSMEG_6758* (encoding an aquaporin), and *MSMEG_6119* (encoding a sulfite exporter) showed dose-dependent upregulation exclusively at the 90-min timepoint.

Among the 86 differentially expressed genes (DEGs) associated with transcriptional regulators, 12 displayed dose-dependent expression (Figure 5B). Genes such as *MSMEG_0663*, *MSMEG_5025* (TetR family), *MSMEG_1953* (WhiB7), *MSMEG_1301* (LuxR family), and *MSMEG_5214* (sigma-70) were upregulated at both timepoints. *MSMEG_0545* (LuxR family) and *MSMEG_5731* (GntR family) were upregulated only at 90 min. Notably, *MSMEG_0663* (TetR family) was the most significantly upregulated and dose-dependent gene at both timepoints. Conversely, four DEGs were downregulated at 90 min: *MSMEG_0330* (LuxR family), *MSMEG_3092* (sugar-binding transcriptional regulator), *MSMEG_6236* (MnoR family), and *MSMEG_5575* (MarR family).

A total of 18 DEGs were associated with acyl-CoA dehydrogenase/oxidase, 12 with enoyl-CoA hydratase/isomerase (Crotonase), one with phytanoyl-CoA dioxygenase, 11 with AMP-dependent synthetase/ligase, and several with CoA-transferase families I and III, all of which are linked to fatty acid degradation and transport. Among these, three DEGs—*MSMEG_2992*, *MSMEG_4547* (acyl-CoA dehydrogenases), and *MSMEG_1419* (phytanoyl-CoA dioxygenase)—were upregulated in a dose-dependent manner at 90 min, while *MSMEG_5348* (long-chain-fatty-acid-CoA ligase) was downregulated at the same timepoint (Figure 5C).

Of the 56 DEGs associated with carbohydrate metabolism and transport, nine were dose-dependently downregulated at 90 min (Figure 5D). These included *MSMEG_5572*, *MSMEG_5574*, *MSMEG_3091*, *MSMEG_4172*, and *MSMEG_6803* (encoding sugar ABC transporters), *MSMEG_5577* (encoding fructokinase), *MSMEG_3092* (encoding a sugar-binding transcriptional regulator), and *MSMEG_3093* and *MSMEG_3095* (encoding FGGY carbohydrate kinases). Notably, *MSMEG_5576* (encoding D-mannonate oxidoreductase) was the most significantly downregulated gene at both timepoints.

Among the 13 DEGs encoding transposases, four—*MSMEG_1728*, *MSMEG_2819*, *MSMEG_5096*, and *MSMEG_2818*—were upregulated in a dose-dependent manner at both timepoints, while *MSMEG_6889* was upregulated only at 90 min.

This comprehensive analysis highlights the dynamic and dose-dependent regulation of genes involved in transport, transcriptional regulation, fatty acid metabolism, carbohydrate metabolism, and transposase activity, providing insights into the molecular responses to the treatment.

#### 2.2.4. LCTA-3368 Induces Reactive Oxygen Species Formation in *M. smegmatis*

To test the hypothesis that the antimicrobial mode of action of LCTA-3368 is based on the formation of reactive oxygen species (ROS), we conducted the 2′,7′-dichlorofluorescein diacetate (H_2_DCF-DA) assay. The conversion of 2′,7′-dichlorodihydrofluorescein diacetate (H_2_DCF-DA) to the highly fluorescent compound 2′,7′-dichlorofluorescein (DCF) occurs in several steps. H_2_DCF-DA is transported across the cell membrane and deacetylated by esterases to form the non-fluorescent 2′,7′-dichlorodihydrofluorescein (H_2_DCF), which becomes trapped inside the cells. H_2_DCF is then oxidized by ROS into fluorescent 2′,7′-dichlorofluorescein (DCF). The fluorescence intensity of DCF reflects the amount of ROS produced [16,17].

**Figure 6:**
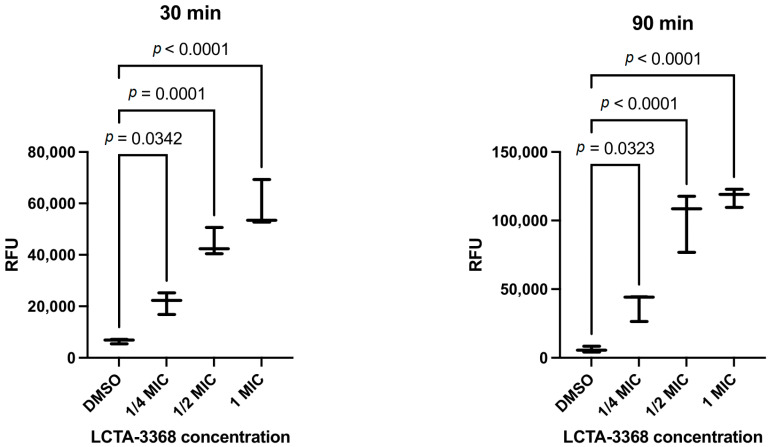
The effect of LCTA-3368 inducing ROS on *M. smegmatis* compared with negative control (DMSO). The left panel represents data for 30 min, while the right one—for 90 min. The Y-axis shows relative fluorescence units (RFU), and the X-axis shows negative control (DMSO) and various concentrations of LCTA-3368. The *p*-values are written above each of the comparison groups. The results with *p*-values of less than 0.05 were considered statistically significant.

The fluorescence of H_2_DCF-DA in *M. smegmatis* intensified in a dose-dependent manner in response to the addition of escalating concentrations of LCTA-3368 (1/4 × MIC to 1 × MIC) at both 30 and 90 min timepoints, indicating an increase in ROS concentration as compared to the negative control (DMSO) as shown in Figure 6.

## 3. Discussion

Currently, there is a pressing need to identify new drug targets and elucidate novel mechanisms of action for combating *Mycobacterium tuberculosis*. Quinoxaline 1,4-di-N-oxides (QdNOs), a class of heterocyclic compounds with N–O groups at the 1- and 4-positions, are known for their reducing potential, which generates free radical intermediates and contributes to their antibacterial activity. QdNOs have demonstrated potent inhibitory activity in vitro against various pathogens, including mycobacteria [4].

In this study, we employed a multi-dose approach with varying levels of inhibition at two timepoints to investigate the early defensive responses of *M. smegmatis* to the QdNO derivative LCTA-3368 at the transcriptomic level. We hypothesized that genes providing protective effects to the bacterial cell would be upregulated while those contributing to the mechanism of action of LCTA-3368 would be downregulated. This hypothesis was confirmed by our detection of an increase in ROS in *M. smegmatis* cells upon exposure to LCTA-3368.

The antibacterial mechanism of QdNOs is often linked to the generation of free radicals during bioreduction, leading to DNA damage [2,14,18,19]. Both *M. smegmatis* and *M. tuberculosis* possess multiple DNA repair pathways, including homologous recombination (HR), nonhomologous end joining (NHEJ), and single-strand annealing (SSA) [20]. In our study, we observed the upregulation of 42 differentially expressed genes (DEGs) involved in DNA repair, with five showing strict dose dependence.

A hallmark of the bacterial response to antibiotic-induced stress is the activation of the SOS repair system, characterized by the increased expression of error-prone DNA polymerases. In our experiment, DNA polymerase III (*MSMEG_1633*) was highly upregulated, along with an error-prone DNA polymerase from the Y family (*MSMEG_1622*), which is known to promote adaptive mutagenesis under stress conditions [21].

Mycobacteria also utilize the nucleotide excision repair (NER) pathway to address DNA damage. This pathway involves the recognition and excision of damaged DNA regions by a complex of UvrA, UvrB, and UvrC proteins, followed by repair synthesis [22,23]. Additionally, crosslink repair DNA glycosylase (*ycaQ*) and apurinic/apyrimidinic endonuclease IV play roles in protecting mycobacterial DNA from oxidative stress [24,25].

Mobile genetic elements (MGEs) further contribute to mutagenesis, with transposases being key drivers of MGE activity. Among the 13 DEGs encoding transposases, five were upregulated in a dose-dependent manner. The increased activity of transposases under drug pressure may accelerate the emergence of drug resistance [26,27].

The broad upregulation in DNA repair systems in our study supports the proposed mechanism of QdNOs involving DNA damage [2,18]. Conversely, the dose-dependent upregulation in mutagenic systems, including error-prone polymerases, the NER pathway, and transposases, aligns with the numerous mutations observed in LCTA-3368-resistant mutants [8].

Oxidoreductases are critical for the bioreductive activation of QdNOs [28]. We identified three oxidoreductase genes (*MSMEG_6763, MSMEG_5576*, and *MSMEG_3094*) that were downregulated in a dose-dependent manner, suggesting their potential role in LCTA-3368 activation. Conversely, eight oxidoreductase genes were upregulated, with three (*MSMEG_1623, MSMEG_4565,* and *MSMEG_5027*) showing the highest levels of differential expression. These oxidoreductases may primarily function to neutralize free radicals generated by LCTA-3368, thereby protecting bacterial cells [28,29,30,31].

In previous work, we identified LCTA-3368-resistant strains with multiple mutations, including those in pyruvate synthase (*MSMEG_4646*), ferredoxin (*MSMEG_5122*), and the transcriptional repressor *MSMEG_1380*, which leads to the overexpression of the MmpS5-MmpL5 efflux system [32]. While we did not observe significant changes in the expression of the MmpS5-MmpL5 system, other multidrug resistance (MDR) transporter genes (*MSMEG_3815, MSMEG_5187, MSMEG_2991*) were overexpressed, indicating their potential role in LCTA-3368 efflux.

Finally, the downregulation in genes involved in lipid and carbohydrate transport, as well as amino acid biosynthesis, suggests a general attenuation of metabolic processes after 90 min of LCTA-3368 treatment, ultimately leading to cell death.

While *M. smegmatis* serves as a useful model, its metabolic differences from M. tuberculosis must be acknowledged. However, most of the dose-dependent DEGs identified in this study have homologs in the *M. tuberculosis* genome (Table S3), suggesting that our findings may be applicable to the pathogen. Further studies on M. tuberculosis, including experiments with customizable media such as variations of the Sauton medium, could provide additional insights into the influence of carbon sources on carbohydrate metabolism and drug efficacy.

## 4. Materials and Methods

### 4.1. Microbial Cultures and Growth Conditions

*Mycobacterium smegmatis* mc2 155 was stored as 10% glycerol stocks at −80 °C. Thawed cultures were plated on Middlebrook 7H11 agar (Himedia, Thane, India) supplemented with oleic albumin dextrose catalase (OADC, Himedia) to single colonies. Soybean-casein digest agar (M290, Himedia) was used as solid media for further culturing on plates, while Middlebrook 7H9 medium (Himedia) supplemented with oleic OADC, 0.1% Tween-80 (v/v), and 0.4% glycerol (v/v) was used as liquid medium. Liquid cultures were incubated in a Multitron incubator shaker (Infors HT, Bottmingen-Basel, Basel, Switzerland) at 37 °C and 250 rpm.

For the drug exposure assay and transcriptomic analysis, *M. smegmatis* mc2 155 was inoculated from agar plates into 7H9 medium and grown until an OD600 of 2.5 (two nights) to obtain a stable liquid culture without clumps. The culture was then diluted 1:200 and grown overnight until reaching an OD600 of 2. Subsequently, it was diluted 1:10 in fresh medium to achieve an approximate OD600 of 0.2. LCTA-3368 100× stock solutions were prepared in DMSO (ACS Grade, Solon, India) and added to the bacterial cultures to final concentrations corresponding to 1/4 × MIC (1 μg/mL), 1/2 × MIC (2 μg/mL), and 1 × MIC (4 μg/mL) in 7H9 OADC medium. The same volume of DMSO (1% v/v) was added to the control samples. Bacterial cultures were incubated for 30 min and 90 min (1/6 and 1/2 of the cell division time, respectively [33]) at 37 °C and 250 rpm, followed by RNA extraction. All experiments were performed in three biological replicates.

### 4.2. Dichloro-Dihydro-Fluorescein Diacetate (H2DCF-DA) Assay

*M. smegmatis* was grown to just before the mid-log phase, as described in the previous experiment. H2DCF-DA 20 mM in DMSO (Lumiprobe, Moscow, Russia) was added to the culture to a final concentration of 10 μM. The final concentration of DMSO is 0.2%. LCTA-3368 was added to the samples at concentrations of 1/4 MIC, 1/2 MIC, 1 MIC, or 0.3% H_2_O_2_ and incubated for 30 min at 37 °C, 250 rpm, in the dark. For the 90-min LCTA-3368 treatment, H2DCF-DA was added 30 min before the end of incubation. Cells were washed twice with PBS (pH 7.4) and centrifuged at 10,000× *g* for 5 min at 4 °C. The cell pellet was lysed with 0.5% Triton X-100 for 10 min at 4 °C in the dark and centrifuged at 12,000× *g* for 10 min at 4 °C. Fluorescence of the supernatant was measured at Ex 485 nm and Em 535 nm. H_2_O_2_ was used as a positive control, and cells grown without treatment (DMSO only) were used as a negative control.

### 4.3. Total RNA Extraction

RNA was extracted using the MagMAX mirVana Total RNA Isolation Kit (Thermo Fisher Scientific, Waltham, MA, USA) on the KingFisher Flex Purification System (Thermo Fisher Scientific), following the manufacturer’s instructions. The extracted RNA was treated with DNase using the Turbo DNA-Free Kit (Thermo Fisher Scientific) in a 50 µL reaction volume and further purified using Agencourt RNAClean XP (Beckman Coulter, Brea, CA, USA) according to the manufacturer’s protocol. The total RNA concentration was measured using the Quant-iT Ribogreen RNA Assay Kit (Thermo Fisher Scientific), and RNA quality was assessed using an Agilent Bioanalyzer with Agilent RNA 6000 Pico Chips (Agilent Technologies, Santa Clara, CA, USA).

### 4.4. Library Preparation and RNA Sequencing

For the preparation of transcriptomic libraries, 250 ng of total RNA was used as input. Ribosomal RNA was selectively removed using the Ribo-Zero Plus rRNA Depletion Kit (Illumina, San Diego, CA, USA), followed by library preparation with the KAPA RNA Hyper Kit (Roche, Basel, Switzerland), according to the manufacturer’s protocol. RNA purification steps were carried out using RNA Clean XP magnetic beads (Beckman Coulter), and final library purification was performed with Agencourt AMPure XP magnetic beads (Beckman Coulter). The size distribution and quality of the libraries were evaluated using the Agilent High Sensitivity DNA Kit (Agilent Technologies), while library concentration was quantified using the Quant-iT DNA Assay Kit, High Sensitivity (Thermo Fisher Scientific). Equimolar quantities of all libraries (12 pM) were pooled and sequenced in a high-throughput run on the Illumina HiSeq platform using 2 × 100 bp paired-end reads and a 5% PhiX spike-in control. The RNA-seq read data were deposited in the NCBI Sequence Read Archive under accession number PRJNA1091547.

### 4.5. Bioinformatics Analysis

Quality control of the raw sequencing data was performed using FastQC (v.0.11.9) [34], and individual reports were merged using MultiQC (v.1.9) [35]. Adapters and low-quality reads were removed with Trimmomatic (v.0.39) [36]. Trimmed reads were mapped to the reference *M. smegmatis* mc^2^ 155 genome (CP000480.1) using HISAT2 (v.2.2.1) [37]. Mapping quality and gene coverage were assessed with QualiMap (v.2.2.2) [38]. Mapped reads were assigned to genes using featureCounts (v.2.0.1) [39]. Differential gene expression (DGE) analysis was conducted using edgeR (v.3.30.3) [40] in R (v.4.0.2) [41]. EdgeR models biological variation through empirical Bayes estimation of dispersions rather than traditional confidence intervals or standard errors for log fold changes (logFC). Standard errors in count-based models (e.g., negative binomial) are unreliable, as demonstrated in the original edgeR paper [40]. EdgeR relies on likelihood ratio tests and exact tests, which are statistically more robust than using standard errors for hypothesis testing. To address biological variability, edgeR estimates gene-wise dispersion values, which are incorporated into differential expression analysis. We have visualized these dispersion estimates in a Biological Coefficient of Variation (BCV) plot (Figure S2), which provides insight into the variance structure of our data. Genes with a false discovery rate (FDR) cutoff of 0.05 and a fold change (FC) of log2FC ≤ −1 or log2FC ≥ 1 were considered differentially expressed. Plots were generated in Python 3 (v.3.13) [42] using the matplotlib (v.3.9) [43] and numpy (v.2.1.1) [44] packages.

Functional enrichment analysis of Gene Ontology (GO) categories and Kyoto Encyclopedia of Genes and Genomes (KEGG) pathways for differentially expressed genes (DEGs) was performed using GOpiscator (v.0.1.5) [45], with categories considered enriched at *p* ≤ 0.05. Heatmaps were generated in Python 3 (v.3.12.6) using the Seaborn (v.0.13) package. Bubble charts based on GOpiscator (v.0.1.5) [45] results for DEGs were created in Python 3 (v.3.12.6) using the matplotlib (v.3.9) and numpy (v.2.1.1) packages.

Statistically significant differences in ROS concentration changes relative to DMSO were calculated using the one-way ANOVA test using the GraphPad Prism program (v.10.4.2) [46]. The results with *p*-values of less than 0.05 were considered statistically significant. Hydrogen peroxide was used as a positive control. Differences in RFU compared to negative control and exposure to LCTA-3368 for 30 and 90 min were statistically significant (Supplementary Figure S3).

## 5. Conclusions

The QdNO compound LCTA-3368 induces oxidative and/or nitrosative stress conditions, triggering a broad transcriptomic response in mycobacterial cells. The primary mechanisms for damage control in these cells involve DNA repair systems, notably the SOS response and nucleotide excision repair (NER). However, these repair processes can introduce mutations, potentially leading to drug resistance. Additionally, genes encoding proteins with oxidoreductase activity exhibit dose-dependent differential expression. This allows for the classification of oxidoreductases into two groups: those involved in activating LCTA-3368 (downregulated) and those potentially capable of deactivating its active metabolites. Our findings pave the way for the targeted synthesis of more specific quinoxaline 1,4-dioxide (QdNO) derivatives.

## Data Availability

Data are contained within the article and Supplementary Materials. Raw sequencing data are available at PRJNA1091547.

## Supplementary Materials

The supporting information can be downloaded at https://www.mdpi.com/article/10.3390/ijms26083689/s1.
