# Supplementary material for: A Quinoxaline 1,4-Dioxide Activates DNA Repair Systems in Mycobacterium smegmatis: A Transcriptomic Study
Source: Int J Mol Sci. 2025 Apr 14;26(8):3689. doi: 10.3390/ijms26083689 (PMC12027616; doi:10.3390/ijms26083689)
Supplement: Supplementary file 1 [file ijms-26-03689-s001.zip › Supplementary Figures.pdf]

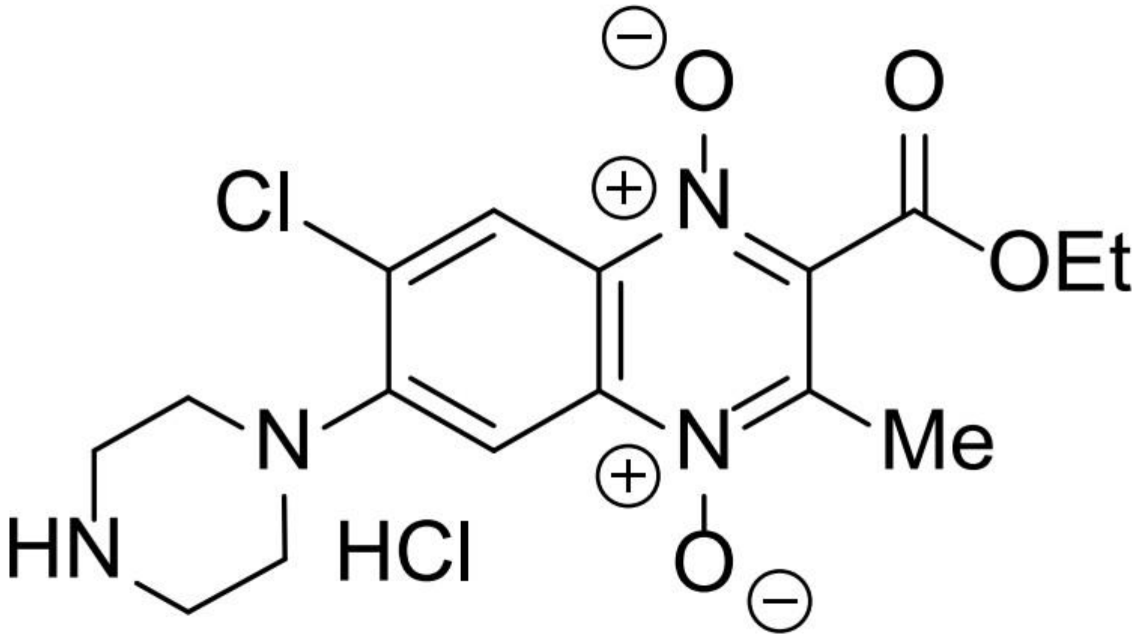

Figure S1. The structural formula of LCTA-3368

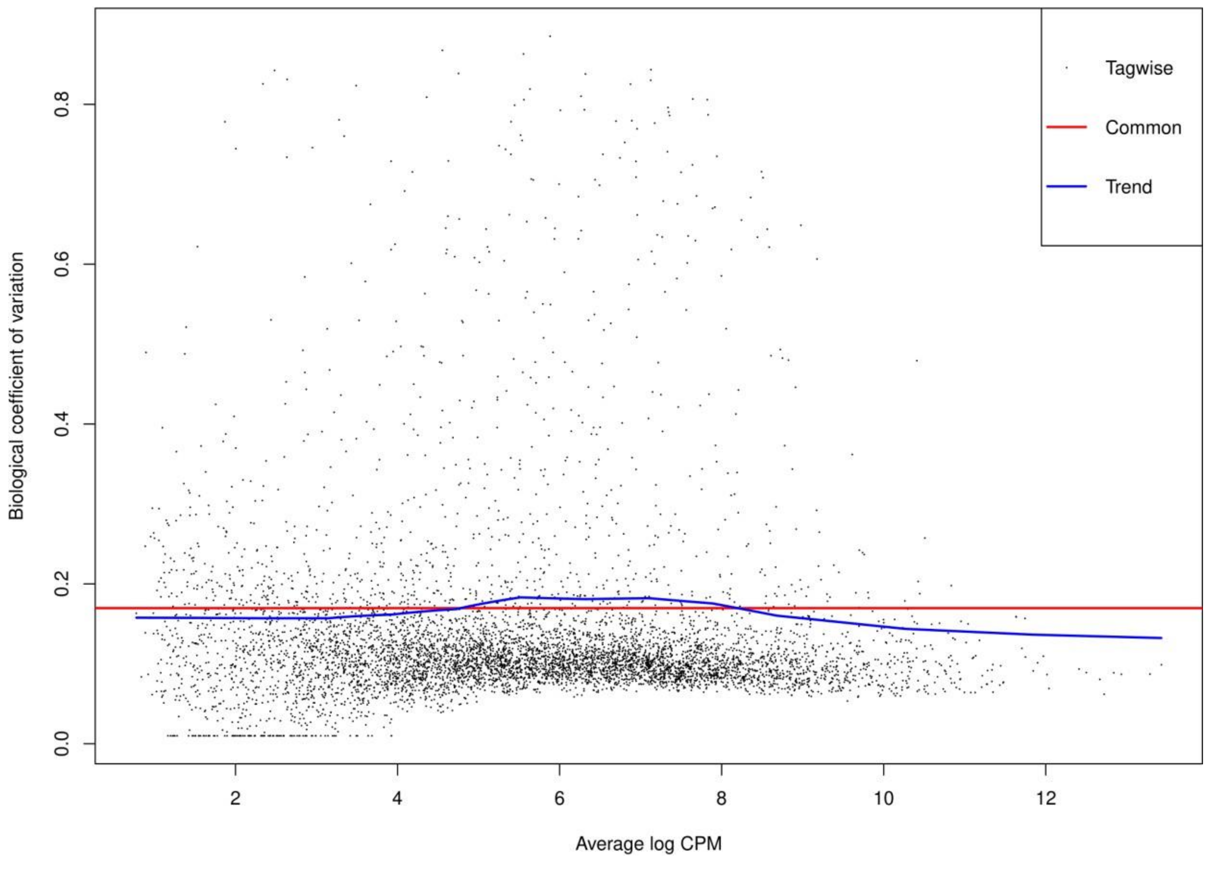

Figure S2. BCV plot providing the variance structure of the data

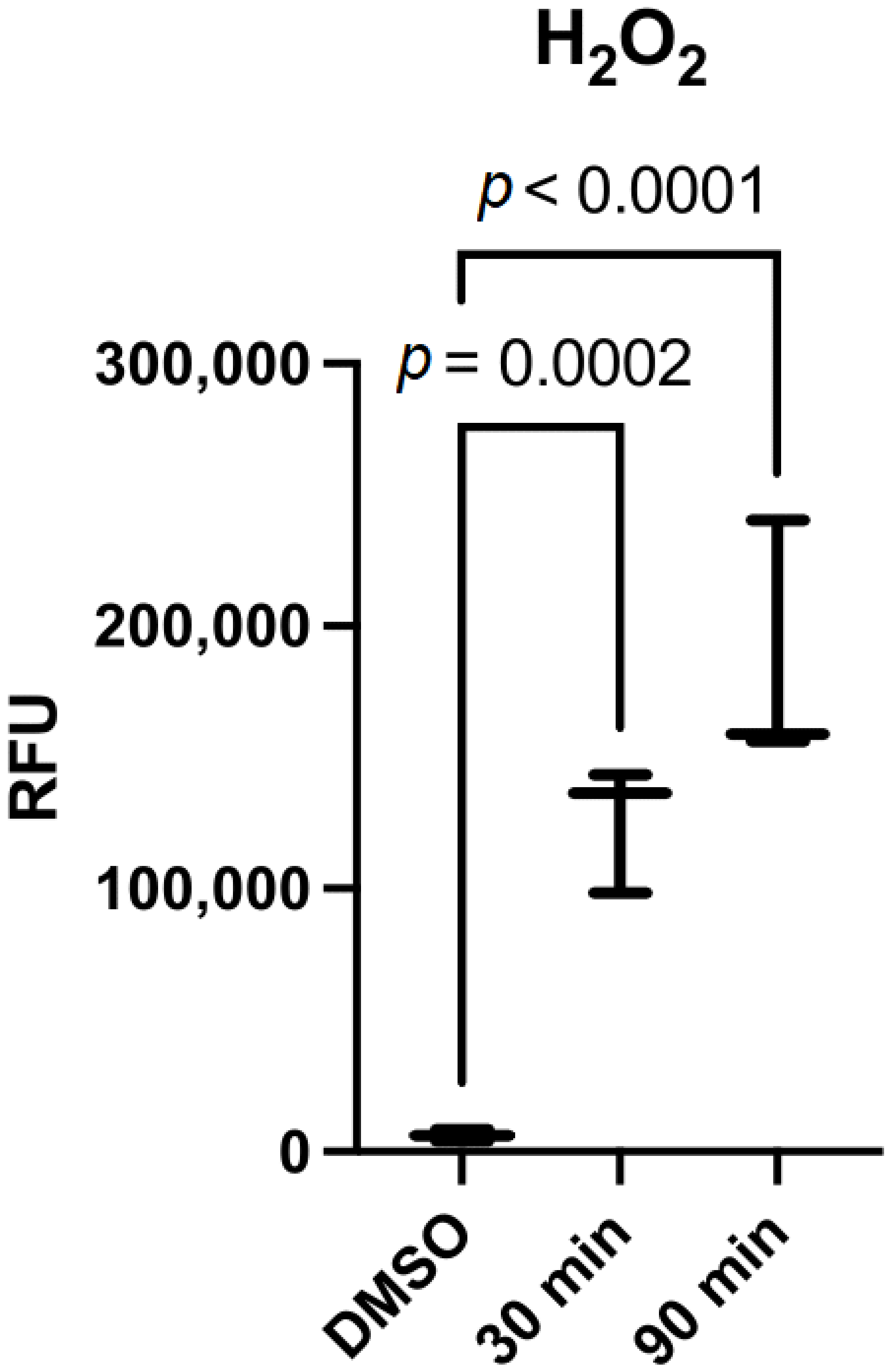

Figure S3. The effects of inducing ROS by H2O2 compared with DMSO and LCTA-3368 for 30 and 90 minutes.
